# Supplementary material for: Characterization and expression analysis of genes encoding three small heat shock proteins in the oriental armyworm, Mythimna separata (Walker)
Source: PLoS One. 2020 Aug 10;15(8):e0235912. doi: 10.1371/journal.pone.0235912 (PMC7417081; doi:10.1371/journal.pone.0235912)
Supplement: S1 Table — (DOC) [file pone.0235912.s001.doc]

**Table S1.** Primers used in this study.

| Primers | Primer sequences | T_m_ (˚C) | Usage |
| --- | --- | --- | --- |
| *Hsp19.7*-F | TGGGGCTRACWCCRGAAGA | 65-45(decreasing 1 ˚C per cycle) | RT-PCR |
| *Hsp19.7*-R | ACCTCCTTGCGMACKGGTCC |  |  |
| *Hsp19.8*-F | AGKGAATACTACAGACCBTGG |  |  |
| *Hsp19.8*-R | TGRTCCTTKATCTCCTTGCG |  |  |
| *Hsp21.4*-F | ATGGARGAAGAAATGASAARTT |  |  |
| *Hsp21.4*-R | GCCTCVGGATTGGTKCCYTTBGG |  |  |
| *Hsp19.7*-3 | ATTTCCCGCCAATTCACTCGCCGCTAC | 68 | RACE |
| *Hsp19.7*-5 | GGCGGGAAATGTAGCCATGTTGGTC | 68 |  |
| *Hsp19.8*-3 | GCGTTGCCAGAGGGTTGTGTGCC | 68 |  |
| *Hsp19.8*-5 | GGCACACAACCCTCTGGCAACGC | 68 |  |
| *Hsp21.4*-3 | GCCACTGGGACAGCCTGAACTCG | 68 |  |
| *Hsp21.4*-5 | GACTTGCCGTCGCCCTCATCCTG | 68 |  |
| *Hsp19.7*-RT-F | TGGCTACATTTCCCGCCAAT | 54.3 | Real-time PCR |
| *Hsp19.7*- RT-R | TGTCACCATTCGCCTGATCC |  |  |
| *Hsp19.8*- RT-F | TGGCCCTAACACCTGACGATG | 58.8 |  |
| *Hsp19.8*- RT-R | ACTTGTCACCGTCAGCCTTGA |  |  |
| *Hsp21.4*- RT-F | ACTAGCAGCACCACATCCTCA | 56.3 |  |
| *Hsp21.4*- RT-R | TGGCTAACATCGAAACGGAGC |  |  |
| *Actin*-F | AACTTCCCGACGGTCAAGTCAT | 60 |  |
| *Actin*-R | TGTTGGCGTACAAGTCCTTACG |  |  |
| *Tub*-F | CGGTAATGCCTGCTGGGAA | 54.3 |  |
| *Tub*-R | CTCGCTGAAGAAGGTGTTGAA |  |  |
