# Supplementary material for: Characterization and expression analysis of genes encoding three small heat shock proteins in the oriental armyworm, Mythimna separata (Walker)
Source: PLoS One. 2020 Aug 10;15(8):e0235912. doi: 10.1371/journal.pone.0235912 (PMC7417081; doi:10.1371/journal.pone.0235912)
Supplement: S2 Table — (DOCX) [file pone.0235912.s002.docx]

| Table S2 Sequences used in phylogenetic analysis | | |
| --- | --- | --- |
| Species name | Protein type | Accession number |
| *Mythimna separata* | Hsp19.7 | MN503276 |
| *Mythimna separata* | Hsp19.8 | MN503277 |
| [*Helicoverpa armigera*](https://www.ncbi.nlm.nih.gov/protein/XP_021195226.1?report=genbank&log$=prottop&blast_rank=1&RID=4HSDDJ87016) | Hsp19.7 | [XP_021195228.1](https://www.ncbi.nlm.nih.gov/protein/XP_021195228.1?report=genbank&log$=prottop&blast_rank=1&RID=4HSSSHXP016) |
| [*Mamestra brassicae*](https://blast.ncbi.nlm.nih.gov/Blast.cgi#alnHdr_BAF03558) | Hsp19.7 | [BAF03558.1](https://www.ncbi.nlm.nih.gov/protein/BAF03558.1?report=genbank&log$=prottop&blast_rank=2&RID=4HSSSHXP016) |
| [*Spodoptera litura*](https://blast.ncbi.nlm.nih.gov/Blast.cgi#alnHdr_XP_022837101) | Hsp19.7 | [XP_022837108.1](https://www.ncbi.nlm.nih.gov/protein/XP_022837108.1?report=genbank&log$=prottop&blast_rank=3&RID=4HSSSHXP016) |
| [*Sesamia inferens*](https://blast.ncbi.nlm.nih.gov/Blast.cgi#alnHdr_AJA32867) | Hsp19.6 | [AJA32867.1](https://www.ncbi.nlm.nih.gov/protein/AJA32867.1?report=genbank&log$=prottop&blast_rank=6&RID=4HSSSHXP016) |
| [*Heliothis virescens*](https://blast.ncbi.nlm.nih.gov/Blast.cgi#alnHdr_PCG66520) | Hsp19.7 | [PCG66520.1](https://www.ncbi.nlm.nih.gov/protein/PCG66520.1?report=genbank&log$=prottop&blast_rank=4&RID=4HSSSHXP016) |
| [*Chilo suppressalis*](https://blast.ncbi.nlm.nih.gov/Blast.cgi#alnHdr_AGC23337) | Hsp19.8 | [AGC23337.1](https://www.ncbi.nlm.nih.gov/protein/AGC23337.1?report=genbank&log$=prottop&blast_rank=23&RID=4HNXGE57016) |
| [*Sesamia nonagrioides*](https://blast.ncbi.nlm.nih.gov/Blast.cgi#alnHdr_ACD01216) | Hsp19.5 | [ACD01216.1](https://www.ncbi.nlm.nih.gov/protein/ACD01216.1?report=genbank&log$=prottop&blast_rank=1&RID=4HU6RKB5016) |
| [*Spodoptera litura*](https://blast.ncbi.nlm.nih.gov/Blast.cgi#alnHdr_XP_022837101) | Hsp20.6 | [XP_022837101.1](https://www.ncbi.nlm.nih.gov/protein/XP_022837101.1?report=genbank&log$=prottop&blast_rank=1&RID=4HNXGE57016) |
| [*Sesamia inferens*](https://blast.ncbi.nlm.nih.gov/Blast.cgi#alnHdr_AJA32865) | Hsp20.6 | [AJA32865.1](https://www.ncbi.nlm.nih.gov/protein/AJA32865.1?report=genbank&log$=prottop&blast_rank=4&RID=4HNXGE57016) |
| [*Helicoverpa armigera*](https://www.ncbi.nlm.nih.gov/protein/XP_021195226.1?report=genbank&log$=prottop&blast_rank=1&RID=4HSDDJ87016) | Hsp20.3 | [XP_021195226.1](https://www.ncbi.nlm.nih.gov/protein/XP_021195226.1?report=genbank&log$=prottop&blast_rank=1&RID=4HSDDJ87016) |
| *Mythimna separata* | Hsp21.4 | MN503278 |
| [*Helicoverpa armigera*](https://www.ncbi.nlm.nih.gov/protein/XP_021195226.1?report=genbank&log$=prottop&blast_rank=1&RID=4HSDDJ87016) | Hsp21.4 | [XP_021184555.1](https://www.ncbi.nlm.nih.gov/protein/XP_021184555.1?report=genbank&log$=prottop&blast_rank=1&RID=4HTATMRM016) |
| [*Sesamia inferens*](https://blast.ncbi.nlm.nih.gov/Blast.cgi#alnHdr_AJA32867) | Hsp21.4 | [AJA32863.1](https://www.ncbi.nlm.nih.gov/protein/AJA32863.1?report=genbank&log$=prottop&blast_rank=2&RID=4HTATMRM016) |
| [*Grapholita molesta*](https://blast.ncbi.nlm.nih.gov/Blast.cgi#alnHdr_AKS40074) | Hsp21.3 | [AKS40074.1](https://www.ncbi.nlm.nih.gov/protein/AKS40074.1?report=genbank&log$=prottop&blast_rank=5&RID=4HTATMRM016) |
| [*Chilo suppressalis*](https://blast.ncbi.nlm.nih.gov/Blast.cgi#alnHdr_AGC23337) | Hsp21.4 | [AGC23338.1](https://www.ncbi.nlm.nih.gov/protein/AGC23338.1?report=genbank&log$=prottop&blast_rank=7&RID=4HTATMRM016) |
| [*Bombyx mori*](https://blast.ncbi.nlm.nih.gov/Blast.cgi#alnHdr_NP_001036985) | Hsp21.4 | [NP_001036985.1](https://www.ncbi.nlm.nih.gov/protein/NP_001036985.1?report=genbank&log$=prottop&blast_rank=4&RID=4HTATMRM016) |
| [*Hyposmocoma kahamanoa*](https://blast.ncbi.nlm.nih.gov/Blast.cgi#alnHdr_XP_026326546) | Hsp21.4 | [XP_026326546.1](https://www.ncbi.nlm.nih.gov/protein/XP_026326546.1?report=genbank&log$=prottop&blast_rank=6&RID=4HTATMRM016) |
| [*Vanessa tameamea*](https://blast.ncbi.nlm.nih.gov/Blast.cgi#alnHdr_XP_026491063) | Hsp21.4 | [XP_026491063.1](https://www.ncbi.nlm.nih.gov/protein/XP_026491063.1?report=genbank&log$=prottop&blast_rank=9&RID=4HTATMRM016) |
