# Supplementary material for: Characterization and expression analysis of genes encoding three small heat shock proteins in the oriental armyworm, Mythimna separata (Walker)
Source: PLoS One. 2020 Aug 10;15(8):e0235912. doi: 10.1371/journal.pone.0235912 (PMC7417081; doi:10.1371/journal.pone.0235912)
Supplement: Attachment — Submitted filename: Responsive letter.docx [file pone.0235912.s003.docx]

**Dear editor:**

According to the comments from you and two reviewers, we revised our manuscripts carefully and made some mofications. More details are as follows:

**Editor comments:**
1. Pg3 Line 5 - the Morrow citation is different from the other citations

Response: We change this citation form according to requirement of Plos one

2. Pg5 LIne 3 - provide more details on how the degenerate primers were designed. Were they designed to a specific region? If so, indicate what that region is. Were they based on previously published primers? If so, provide the appropriate reference.

Response: We described the degenerate primers information in detail, please see page 5 line3-5

3. Pg 5 Line 19 - Perhaps replicate with a more robust analysis such as maximum likelihood and include an outgroup to root the tree.

Response: Phylogenetic trees were re-constructed by MEGA v. 7 using the maximum likelihood method with 2000 bootstrap replicates and the sHsps of *D.melanogaste* were used as outgroup.

4. Pg 6 LIne 6 - Were amplification efficiencies determined and factored into the analysis?It is widely accepted that studies presenting qRT-PCR data should follow the MIQE guidelines (see Bustin et al. 2009; Clinical Chemistry 55:611–622).

Response: The amplification efficiencies were determined and factored into the analysis.

5. Pg 6 Line 18 - Indicate that the respective sHsps were named/defined based on the predicted MW.

Response: We indicated the respective sHsps were named/defined based on the predicted MW.

6. Pg 6 - Was BLAST analysis of the predicted proteins done? If so, a Table or Supplementary Table could be included to indicate the top hits.

Response: We conducted BLAST analysis of the predicted proteins , and indicated the thop hits , please see page page6 line1-6.

7. Pg 7 Line 7 - the phylogenetic results support their annotation as sHsps they do not confirm this.

Response: We confirmed this restults.

8. Pg 8 Line 11 states that the three sHsps had similar expression patterns in epidermis regardless of the phase. However, the data shown in figure 4 indicate that sHsp21.4 is higher in gregarious, whereas the other two sHsps are higher in solitary epidermis. Please clarify.

Response: We clarify the expresson patterns of three sHsps, please see in page8 line17-22.

9. Pg 11 Line 14 - the proposal that the sHsps contribute to improved resistance is highly speculative. Unless there is prior data (which should be cited and explicitly described) indicating that gregarious phase M. separata support this assertion, then this would be better described as a hypothesis worth testing in future studies.

Response: We described it as a hypothesis worth testing in future studies
**Reviewer #1:**

This study investigated three small Hsps in the species the oriental armyworm. The authors cloned the three genes, analyzed the sequences and performed expression analysis in different developmental stages and tissues as well as in different population density. The paper provides useful information on the expresson of the three small Hsps in response to developmental and density changes. I have the following concerns that needs be addressed by the authors:
1. In Fig. 1, α-crystallin domain should be conserved. However, as shown in this figure, Hsp21.4 presents non-conseved sequence with the other two small Hsps. Please clarify it by comparing with the conserved crystallin domain.

Response: We clarified the α-crystallin domainof three sHsps by multiple comparion.
2. The relative mRNA levels of small Hsps between gregarious and solitarious forms varied with developmental stages and tissues. The authors should discuss the results and give explanations.

Response: In fact, in our original manuscript,we have discussed the results and give possible reasons,more details in page page 9-10.
3. The following sentence in Abstract is not exactly stated: "Real-time PCR analyses revealed that the three sHsps were transcribed in all developmental stages and were dramatically up-regulated at the 6th larval stage in gregarious individuals." Only hsp19.7 but not other small hsps is dramatically upregulated at the 6th stage.

Response: we cheked the expression patterns throughout the developmental stage, all three hsps were dramatically up-regulated at the 6th larval stage in gregarious individuals compared to that of solitary ones.
**Reviewer #2:**

I am confused with the key point. Whether population density induced Hsp expression or Hsp up/down expression induced gregarious and solitary phases? Rooting a phylogenetic tree with an outgroup organism or sequence will be better. And latest version of Mega, too. By the way, consecutive numbering will be better for a reviewer.
**Page 2**
Line 14: change “Orthopteran, Lepidopteran, Hemipteran and Coleopteran species” to “orthopterans, lepidopterans, hemipterans and coleopterans”

Response: We changed “Orthopteran, Lepidopteran, Hemipteran and Coleopteran species” to “orthopterans, lepidopterans, hemipterans and coleopterans”
Line 17: change “than solitary forms” to” than that of solitary forms”

Response: We added “that of”
Line 23: one of, delete

Response: “One of” was deleted
**Page 3**
Line 7: Unfortunately, delete

Response: “Unfortunately” was deleted.
Line 8: abiotic stress, explain in detail

Response: We give the abiotic stress in detail, including extreme temperature, UV irradiation, oxidation, chemicals expsoure, etc.
Lines 12-14, 16-18: reorganize the sentence

Response: We reorganized this sentence.
**Page 4**
Line 3: solitary individuals, how many?

Response: The number of solitary individuals is 1 neonates/container, and the volume of the container is 300 mL.
Line 4: as described, explain in detail

Response: the raised method was given in our published paper (Li et al., 2019, J

Econ Entomol), so we have cited this reference.
Lines 8-10: sample size

Response: the sample size was the same as our published paper(Li et al., 2019. Plos one), we have cited this reference
Line 20: PCR primers

Response: We have give the primers.
**Page 5**
Line 14: “ORF”, what’s this?

Response: “ORF” is the abbreviation of “ open reading frames”.
Line 19: “MEGA v. 5”, why “v. 5”, why not use the latest version?

Response: We used MEAG v.7 to generate the phylogenetic tree

**Page 6**
Lines 1-3: total volume is not 20 μL.

Response: we checked the composition(SsoAdvanced Universal SYBR Green Supermix (10 μL, Bio-Rad), gene-specific primers (1 μL each, Table S1), cDNA template (1 μL), and ddH2O (7 μL)), and the volume was 20μL.
Line 13-14: DPS software, which procedure?

Response: We added the procedure( Scientific Press, China) in the methods.
Line 23: “isoelectric points”, predicted isoelectric points

Response: we added the word”predicted”.
**Page 7**
Line 1: Rooting a phylogenetic tree with an outgroup organism or sequence will be better. How to build the phylogenetic tree should be mentioned in detail in the methods section.

Response: We give the procedure of building the phylogenetic tree in detaile, please see page5 line20-21.
Lines 6-8: “HaHsp”, what’s this?

Response: Hsps in Helicoverpa armigera.
Line 12: the 2nd larvae looks different, why? Explain more in discussion.
Response: Expression of three sHsps failed to show any consistent trend before the fifth-instar larvae. We think that a possible explanation for such expression is that the body size in these developmental stages is so small that each individual may occupy

enough space to lower the mean contact rate between individuals
**Page 9**
Line 6: “in Noctuidae species”, in other Noctuidae species
Response: We added the word”other”
**Page 10**
Lines 3-15: why these tissues? Which one is the key tissue? And why?

Response: We think these tissues include the most tissues. To our knowledge, we think head, epidemics and guts are key tissues. First, the head is the most important tissue/organs in sensing environmental stress signals. Secondly, the epidemics is an important tissue when the gregarious ones contact under high density conditions. Thirdly, the guts play impornt role in absorbing food and protecting from injury.
